# Polyunsaturated fatty acid receptors, GPR40 and GPR120, are expressed in the hypothalamus and control energy homeostasis and inflammation

**DOI:** 10.1186/s12974-017-0869-7

**Published:** 2017-04-26

**Authors:** Nathalia R. V. Dragano, Carina Solon, Albina F. Ramalho, Rodrigo F. de Moura, Daniela S. Razolli, Elisabeth Christiansen, Carlos Azevedo, Trond Ulven, Licio A. Velloso

**Affiliations:** 1 Laboratory of Cell Signaling and Obesity and Comorbidities Research Center, University of Campinas, Campinas, SP 13084-970 Brazil; 2 Department of Physics, Chemistry and Pharmacy, University of Southern Denmark, DK-5230 Odense, Denmark; 3 Laboratory of Cell Signaling, University of Campinas, Rua Cinco de Junho, 350, Cidade Universitária, Campinas, SP 13083-877 Brazil

**Keywords:** GPR40, GPR120, Obesity, Hypothalamic inflammation

## Abstract

**Background:**

The consumption of large amounts of dietary fats is one of the most important environmental factors contributing to the development of obesity and metabolic disorders. GPR120 and GPR40 are polyunsaturated fatty acid receptors that exert a number of systemic effects that are beneficial for metabolic and inflammatory diseases. Here, we evaluate the expression and potential role of hypothalamic GPR120 and GPR40 as targets for the treatment of obesity.

**Methods:**

Male Swiss (6-weeks old), were fed with a high fat diet (HFD, 60% of kcal from fat) for 4 weeks. Next, mice underwent stereotaxic surgery to place an indwelling cannula into the right lateral ventricle. intracerebroventricular (icv)-cannulated mice were treated twice a day for 6 days with 2.0 μL saline or GPR40 and GPR120 agonists: GW9508, TUG1197, or TUG905 (2.0 μL, 1.0 mM). Food intake and body mass were measured during the treatment period. At the end of the experiment, the hypothalamus was collected for real-time PCR analysis.

**Results:**

We show that both receptors are expressed in the hypothalamus; GPR120 is primarily present in microglia, whereas GPR40 is expressed in neurons. Upon intracerebroventricular treatment, GW9508, a non-specific agonist for both receptors, reduced energy efficiency and the expression of inflammatory genes in the hypothalamus. Reducing GPR120 hypothalamic expression using a lentivirus-based approach resulted in the loss of the anti-inflammatory effect of GW9508 and increased energy efficiency. Intracerebroventricular treatment with the GPR120- and GPR40-specific agonists TUG1197 and TUG905, respectively, resulted in milder effects than those produced by GW9508.

**Conclusions:**

GPR120 and GPR40 act in concert in the hypothalamus to reduce energy efficiency and regulate the inflammation associated with obesity. The combined activation of both receptors in the hypothalamus results in better metabolic outcomes than the isolated activation of either receptor alone.

**Electronic supplementary material:**

The online version of this article (doi:10.1186/s12974-017-0869-7) contains supplementary material, which is available to authorized users.

## Background

The consumption of large amounts of dietary fats is one of the most important environmental factors contributing to the development of obesity and metabolic disorders [1–3]. Long-chain saturated fatty acids trigger inflammation through the activation of toll-like receptor-4 and the induction of endoplasmic reticulum stress [4–6]. The low-intensity inflammation generated in this context can act both systemically and on selected anatomical regions to affect insulin and leptin actions [7], insulin production [8, 9], lipid metabolism [10], blood pressure [11], longevity [12], and a number of other parameters involved in the regulation of whole-body energy homeostasis.

Because of the role played by metabolic inflammation in the pathogenesis of insulin and leptin resistance, it has been proposed that approaches leading to the attenuation of inflammation could have a beneficial impact on conditions such as obesity, type 2 diabetes, dyslipidemia, and hypertension [13–15]. In fact, genetic and pharmacological approaches aimed at reducing inflammation have produced encouraging outcomes in various experimental models [9, 14]. In addition, a recent clinical trial targeted the inhibitor of kappa kinase (IKK) using salsalate, resulting in a significant reduction of glycated hemoglobin levels in patients with type 2 diabetes [16].

Studies have identified the polyunsaturated fatty acid (PUFA) receptors GPR120 and GPR40 as attractive potential targets for the treatment of insulin resistance [17–20]. The activation of GPR120 by PUFAs or synthetic ligands engages an atypical signaling system that attenuates metabolic inflammation in obesity and type 2 diabetes [21]. A recent study has reported the beneficial effect of a synthetic agonist of GPR120 in improving glucose intolerance and hepatic steatosis in an animal model of diet-induced obesity [22]. In addition, a number of studies have reported the potential benefits of systemically targeting GPR40 in type 2 diabetes [23, 24].

A great advance in the field was achieved in 2009 when Oh and co-workers [18] described a completely new anti-inflammatory mechanism involving the action of PUFAs through GPR120. Upon ligand binding, GPR120 recruits β-arrestin-2, leading to the internalization of the receptor/regulatory protein complex. The internalized β-arrestin-2 binds to TAB1 and inhibits its binding to TAK1, which results in the inhibition of its activity. TAK1 is a point of convergence for TNF-α and TLR4 signal transduction, and its inhibition impairs the progression of the signal toward JNK and IKK activation, which results in the inhibition of inflammation [18]. In a recent study, the same group reported beneficial metabolic effects of a small molecule that acts as a specific agonist for GPR120 [22]. Obese mice treated with this molecule exhibit improved glucose tolerance and decreased hepatic steatosis accompanied by a reduction of the metabolic inflammation phenotype, indicating GPR120 is an attractive potential target for the treatment of obesity-associated metabolic disorders.

A number of studies have also shown the beneficial metabolic effects of GPR40 activation [20, 25, 26]. This receptor is highly expressed in pancreatic β-cells and, upon activation by PUFAs, it increases glucose-induced insulin secretion [20]. In addition, it has been shown that GPR40 expressed in intestinal L and K cells induces GLP1 and GIP secretion, providing yet another stimulus for insulin secretion [27]. Although the mechanisms of action of GPR40 are less understood than those of GPR120, studies have shown that the induction of Ca^++^ mobilization and activation of CREB may play important roles in some of the effects of this pathway [28]. Thus, the potential therapeutic usefulness of agonists for GPR40 is considered relevant [24].

As an attempt to advance understanding of the mechanisms underlying the beneficial metabolic effects of the activation of GPR120 and GPR40, we decided to evaluate their expression and function in the hypothalamus. We show that GPR120 was predominantly expressed in microglia, whereas GPR40 was predominantly expressed in neurons. Furthermore, their activation reduced obesity-associated hypothalamic inflammation and reduced whole-body energy efficiency.

## Methods

### GPR120 and GPR40 synthetic agonists

GW9508 was purchased from Tocris Bioscience (Ellisville, MO, USA). TUG905 [29] and TUG1197 [30] were synthesized as previously described.

### Chemicals and reagents

All of the reagents for SDS-polyacrylamide gel electrophoresis and immunoblotting were from Bio-Rad (Richmond, CA, USA). HEPES, phenylmethylsulfonyl fluoride, aprotinin, dithiothreitol, Triton X-100, Tween 20, glycerol, and bovine serum albumin (fraction V) were purchased from Sigma Chemical Co. (St. Louis, MO, USA). The antibodies against GPR120 (sc99105), GPR40 (sc32905), neuropeptide-Y (NPY) (sc133080), proopiomelanocortin (POMC) (sc18263), Iba1 (sc28530), β-arrestin-2 (sc13140), vimentin (sc373717), insulin-like growth factor binding protein-2 (IGFBP2) (sc365368), and p-IKKα/β (Thr 23) (sc21660) were from Santa Cruz Biotechnology (Santa Cruz, CA, USA). The α-tubulin (T5168) antibody was from Sigma-Aldrich (St. Louis, MO, USA). The antibodies against mannose receptor (ab8918) and beta-actin (ab8227) were from Abcam (Cambridge, MA, USA). The reagents for chemiluminescence labeling of proteins in immunoblots were from Amersham (Aylesbury, UK). Fluorescein-isothiocyanate (FITC)-conjugated anti-rabbit (sc2012), FITC-conjugated anti-goat (sc2024), rhodamine-conjugated anti-rabbit (sc2091), and rhodamine-conjugated anti-goat (sc094) antibodies were from Santa Cruz Biotechnology (Santa Cruz, CA, USA). Reagents for real-time polymerase chain reaction (PCR) analysis were from Invitrogen (Carlsbad, CA, USA) and Applied Biosystems (Foster City, CA, USA). Primers for tumor necrosis factor-alpha (TNF-α) (Mm00443258_m1), interleukin-1 beta (IL1β) (Mm00434228_m1), interleukin-6 (IL6) (Mm00446190_m1), interleukin-10 (IL10) (Mm01288386_m1), GPR120 (Mm00726193_m1), GPR40 (Mm00809442_s1), peroxisome proliferator activator gamma coactivator 1 alpha (PGC1α) (Mm00447183_m1), uncoupling protein-1 (UCP1) (Mm01244861_m1), cytochrome c (Mm01621044_g1), and glyceraldehyde-3-phosphate dehydrogenase (GAPD) (#4352339E) were obtained from Applied Biosystems.

### Experimental animals

Male Swiss mice originally imported from the Jackson Laboratory and currently bred at the University of Campinas Breeding Center were used in the study. The animals were maintained at 21 ± 3 °C on a 12-h artificial light/dark cycle and were housed in individual cages. At the fifth week of life, mice were randomly assigned to either a standard rodent chow diet (Chow) or high-fat diet (HFD, 60% of energy value from fat, Table 1, at the end of the document text file.) for 4 weeks or 10 weeks, depending on the protocol.

**Table 1:** Macronutrient composition of diets

	Chow diet		HFD	
Macronutrients	g/100 g	Kcal%	g/100 g	Kcal%
Protein	20	80	20	80
Carbohydrates	62	248	45	180
Lipids	4	36	35	315
Kcal/100 g		364		575

### Experimental protocols

For the evaluation of GPR120 and GPR40 expression in the white adipose tissue, liver, intestine, frontal cortex, occipital cortex, hippocampus, and hypothalamus, mice were fed for 9 weeks on chow and then employed for RNA extraction. To assess GPR120 signal transduction, mice fed a HFD for 4 weeks were anesthetized and subjected to acute lateral ventricle hypothalamic microinjection of 2.0 μL of vehicle or GW9508 (1.0 mM), using a Stoelting stereotaxic apparatus set on the following coordinates: anteroposterior, 0.34 mm; lateral, 1.0 mm; and depth, 2.2 mm. After 90 min, the hypothalamus was removed, and protein or RNA extracts were utilized in immunoprecipitation and western blot experiments or real-time PCR analysis, respectively. In another set of experiments, mice were randomly assigned to chow or a HFD for 4 weeks; subsequently, mice were positioned in a Stoelting stereotaxic apparatus to place a cannula in the lateral ventricle using the stereotaxic coordinates described above. Correct positioning of the cannula was tested 5 days after cannulation by evaluation of the thirst response elicited by intracerebroventricular (icv) angiotensin II (10^−6^ M). Icv-cannulated mice were treated twice a day for 6 days with 2.0 μL of vehicle, GW9508 (1.0 mM), TUG1197 (1.0 mM), or TUG905 (1.0 mM). Treatments were administered at 8 am and 5 pm. Food intake and body mass were measured during the treatment period. In each group, some mice were randomly selected for indirect calorimetry and spontaneous physical activity measurements. At the end of the experiment, the hypothalamus was collected for real-time PCR analysis.

### Cell culture

The neuronal cell line mHypoA 2/29 CLU189 (CLU189) and the microglial cell line BV2 were cultivated to 70% confluence in Dulbecco’s modified Eagle’s medium (DMEM) containing 25 mM glucose and 10% fetal bovine serum. Both cell lines were treated with GW9508 (100 μM), TUG1197 (100 μM), and TUG905 (100 μM) for 1 h prior to lipopolysaccharide (LPS) (100 ng/mL) treatment for 10 min and then subjected to western blot analysis. The doses of the agonists were selected following a dose response experiment.

### Lentivirus efficiency

Five commercially available shRNA-encoding lentivirus plasmids to GPR120: LV1 (NM_181748 1-123s1c1), LV2 (NM_181748 1-816s1c1), LV3 (NM_181748 1-852s1c1), LV4 (NM_181748 1-1105s1c1), and LV5 (NM_181748 1-1253s1c1), and a scrambled shRNA control plasmid (SHC016V 11051235 MN) (Sigma-Aldrich) were tested in the CLU189 cell line to determine the effectiveness of each shRNA-mediated GPR120 knockdown. GPR120 silencing was evaluated in cell lysates by western blot analysis.

### Hypothalamic lentivirus delivery

Hypothalamic delivery of the lentivirus was performed in Swiss mice after 4 weeks on a HFD. Mice were placed in a Stoelting stereotaxic apparatus for bilateral hypothalamic injection of 1.0 μL of lentivirus using a Hamilton syringe linked to a 30-gauge needle. The coordinates were adjusted to target the arcuate nucleus as follows: anteroposterior, −1.7 mm; lateral, 0.3 mm; dorsoventral, −5.5 mm. After the lentivirus infection, body mass and food intake were recorded over 10 weeks. In the eigth and ninth experimental weeks, the animals were subjected to an intraperitoneal glucose tolerance test and an intraperitoneal insulin tolerance test. At the end of the tenth experimental week, a cannula was placed in the lateral ventricle (as previously described), and the mice were treated twice a day for 6 days with 2.0 μL of vehicle or GW9508 (1.0 mM). Finally, the hypothalamus was collected for analysis.

### Intraperitoneal glucose tolerance test (ipGTT) and insulin tolerance test (ipITT)

An Intraperitoneal glucose tolerance test (ipGTT)and an insulin tolerance test (ipITT) were performed on food-deprived (6 h) non-anesthetized mice. Blood glucose levels were measured with an OptiumTM mini (Abbott Diabetes Care, Alameda, CA, USA) handheld glucometer using appropriate test strips. For the ipGTT, a solution of 20% glucose (2.0 g/kg body weight) was administered into the peritoneal cavity. Blood samples were collected from the tail vein at 30, 60, 90, and 120 min post-glucose administration for the determination of glucose concentrations. The area under the curve (AUC) was calculated using these values. For the ipITT, glucose blood levels were sampled 5, 10, 15, 20, 25, and 30 min following the intraperitoneal injection of insulin (0.75 U/kg). The rate constant for glucose disappearance during the insulin tolerance test (Kitt) was calculated using the formula 0.693/t1/2. The t1/2 for glucose was calculated from the slope of the least-squares analysis of the plasma glucose concentrations during the linear decay phase [31].

### Western blot and immunoprecipitation

Hypothalamic specimens were homogenized, and samples containing 75–100 mg protein were used as a whole-tissue extract in western blot experiments, as previously described [31]. The proteins were separated by sodium dodecyl sulfate-polyacrylamide gel electrophoresis (SDS-PAGE) transferred to nitrocellulose membranes and blotted with the antibodies indicated, as described in the Results. Specific bands were labeled by chemiluminescence and were quantified by optical densitometry. For immunoprecipitation, hypothalamic lysates (500 μg protein content) were incubated with 1 mg of anti-β-arrestin-2 antibody overnight at 4 °C, and immune complexes were precipitated with Protein A for 6 h. The samples were then washed with phosphate-buffered solution and were resuspended in sample buffer. Immune complexes were separated by SDS-PAGE, and membranes were blotted with either GPR120 or β-arrestin-2 antibodies.

### RNA extraction and quantitative real-time PCR

Total RNA was extracted using the commercially available acid-phenol reagent Trizol (Invitrogen Corp.). RNA concentration, purity, and integrity were confirmed spectrophotometrically using a Nanodrop (ND-1000; Nanodrop Technologies, Wilmington, DE). The first-strand cDNA was synthesized using SuperScript III reverse transcriptase and random hexamer primers as described in the manufacturer’s protocol (Invitrogen Corp.). Quantitative PCR was run to determine the expression of TNF-α, IL1β, IL6, IL10, POMC, and NPY in the hypothalamus and to determine the expression of PGC1α, UCP1 and cytochrome c in the brown adipose tissue using primers supplied with commercially available assays from Applied Biosystems. The reference gene was GAPD. Real-time PCR analysis of gene expression was carried out in an ABI Prism 7500 sequence detection system (Applied Biosystems). For each gene, the optimal concentration of complementary DNA and primers as well as the maximum efficiency of amplification were obtained through 5-point, twofold dilution curve analysis. Amplification was performed in a 20-μL final volume containing 40–50 ng of reverse-transcribed RNA according to the manufacturer’s recommendations using the TaqMan PCR master mix. Real-time data were analyzed using the Sequence Detector System 1.7 (Applied Biosystems). The results are expressed as relative transcript amounts, as previously optimized [32].

### Immunofluorescence staining

For histological analysis, deep-frozen hypothalamic tissue samples were employed in coronal sectioning (5.0 μm). Specimens were double-labeled with either anti-GPR120 or anti-GPR40 antibodies and specific primary antibodies against markers related to the different cell types, including NPY, POMC, Iba1, mannose receptor, vimentin, and IGFBP2. Thereafter, the sections were incubated with specific FITC- or rhodamine-conjugated IgG secondary antibodies and DAPI for nuclear staining. Immunofluorescence imaging was performed to evaluate the distribution of GPR120 and GPR40 in the hypothalamus of the mice. The specificity of the antibodies against GPR120 and GPR40 was evaluated in a separate set of experiments (Additional file 1: Figure S1).

### Indirect calorimetry and spontaneous physical activity

Oxygen consumption/carbon dioxide production and spontaneous physical activity were measured in fed animals using a computer-controlled, open-circuit calorimeter system (LE405 gas analyzer; Panlab-Harvard Apparatus). Mice were single-housed in clear respiratory chambers, and room air passed through the chambers at a flow rate of ten times the body weight of each animal. The air-flow within each chamber was monitored by a sensor (Air Supply and Switching; Panlab-Harvard Apparatus). Gas sensors were calibrated prior to the onset of experiments with primary gas standards containing known concentrations of O_2_, CO_2_, and N_2_ (Air Liquid). The analyses were performed over a 24-h period. Outdoor air reference values were sampled after every four measurements. Sample air was sequentially passed through O_2_ and CO_2_ sensors for the determination of O_2_ and CO_2_ content, from which the measures of oxygen consumption (VO_2_) and carbon dioxide production (VCO_2_) were estimated. The VO_2_ and VCO_2_ were calculated by Metabolism version 2.2 software based on the Withers equation and are expressed in milliliters per hour-1 per gram-1. The respiratory quotient was calculated as VCO_2_/VO_2_. Energy expenditure was estimated as VO_2_/body mass (grams) [33].

### Statistical analysis

All results are reported as the mean ± SEM. Differences between the treatment groups were evaluated using an unpaired Student’s *t* test or a one-way analysis of variance (ANOVA). When the ANOVA indicated significance, a Tukey-Kramer post-hoc test was performed (GraphPad Software, San Diego, CA, USA). A *p* < 0.05 was accepted as statistically significant.

## Results

### The expression of GPR120 and GPR40 in the hypothalamus of mice

**Fig. 1:**
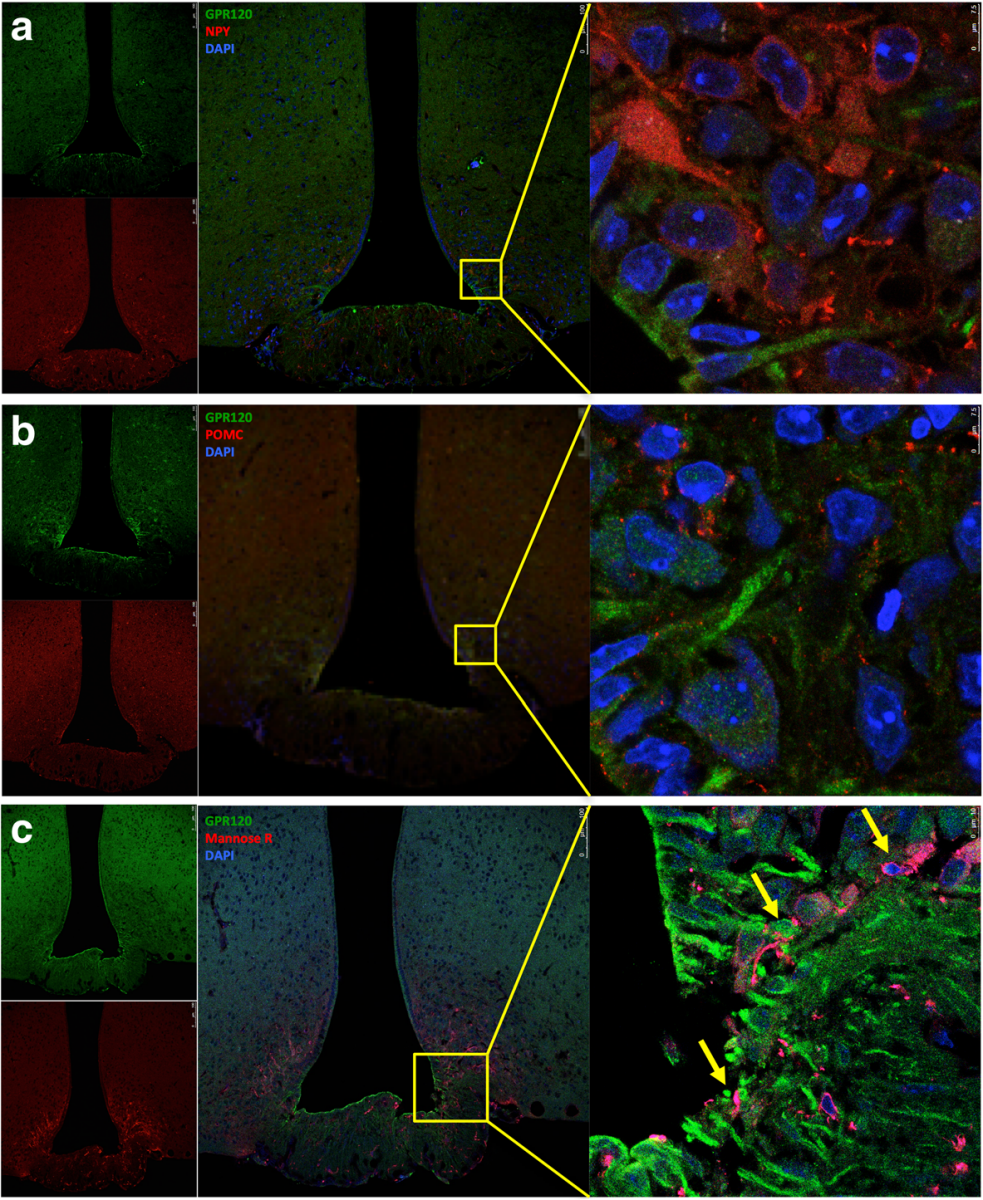
Cellular distribution of GPR120 in the hypothalamus of mice. Tissue sections (5.0 μm) were prepared from the hypothalamic region of lean Swiss mice and were evaluated by indirect immunofluorescence staining using antibodies against GPR120 (**a–c**, *green*), NPY (**a**, *red*), POMC (**b**, *red*), and mannose receptor (**c**, *red*). Nuclei were stained with DAPI (*blue*). In the captions, the *arrows* indicate cells co-expressing GPR120 and mannose receptor (**c**). Images are representative of three independent experiments

**Fig. 2:**
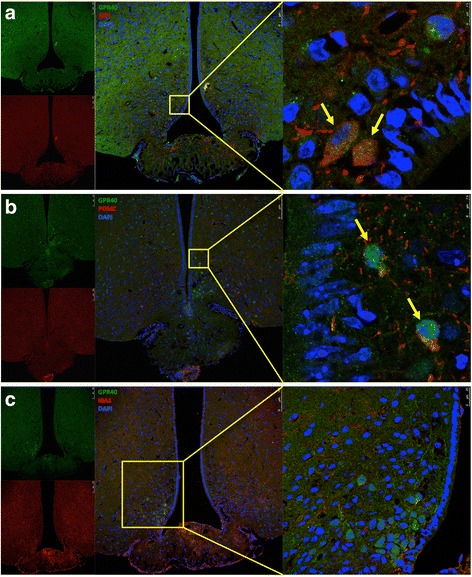
Cellular distribution of GPR40 in the hypothalamus of mice. Tissue sections (5.0 μm) were prepared from the hypothalamic region of lean Swiss mice and were evaluated by indirect immunofluorescence staining using antibodies against GPR40 (**a–c**, *green*), NPY (**a**, *red*), POMC (**b**, *red*), and Iba1 (**c**, *red*). Nuclei were stained with DAPI (*blue*). In the captions, the *arrows* indicate cells co-expressing GPR40 and NPY (**a**), and GPR40 and POMC (**b**). Images are representative of three independent experiments

**Fig. 3:**
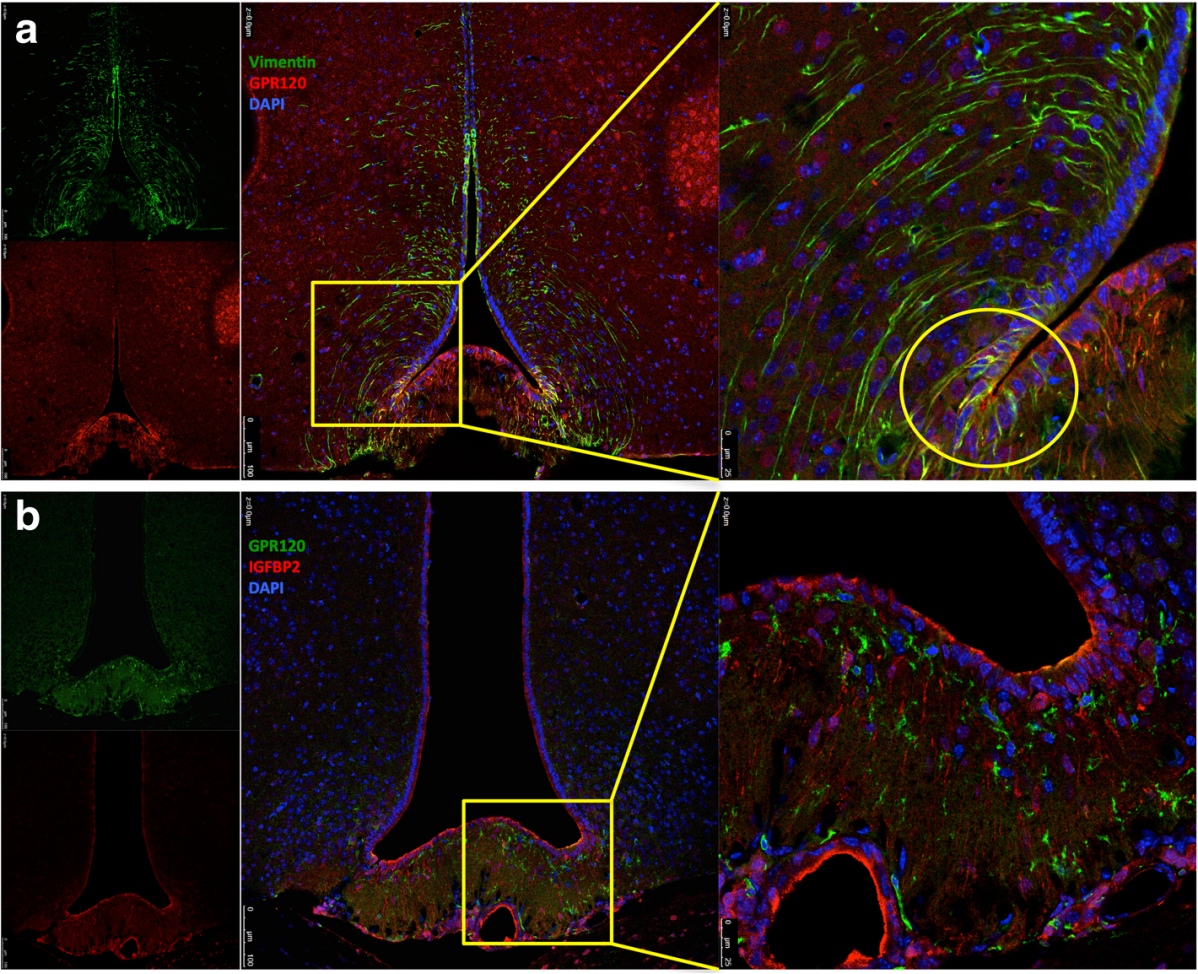
Cellular distribution of GPR120 in the hypothalamus of mice. Tissue sections (5.0 μm) were prepared from the hypothalamic region of lean Swiss mice and were evaluated by indirect immunofluorescence staining using antibodies against GPR120 (**a–b**, *red* in *A* and *green* in *B*), vimentin (**a**, *green*), and IGFBP2 (**b**, *red*). Nuclei were stained with DAPI (*blue*). In the captions, the *yellow circle* indicates region with cells co-expressing GPR120 and vimentin (**a**). Images are representative of three independent experiments

**Fig. 4:**
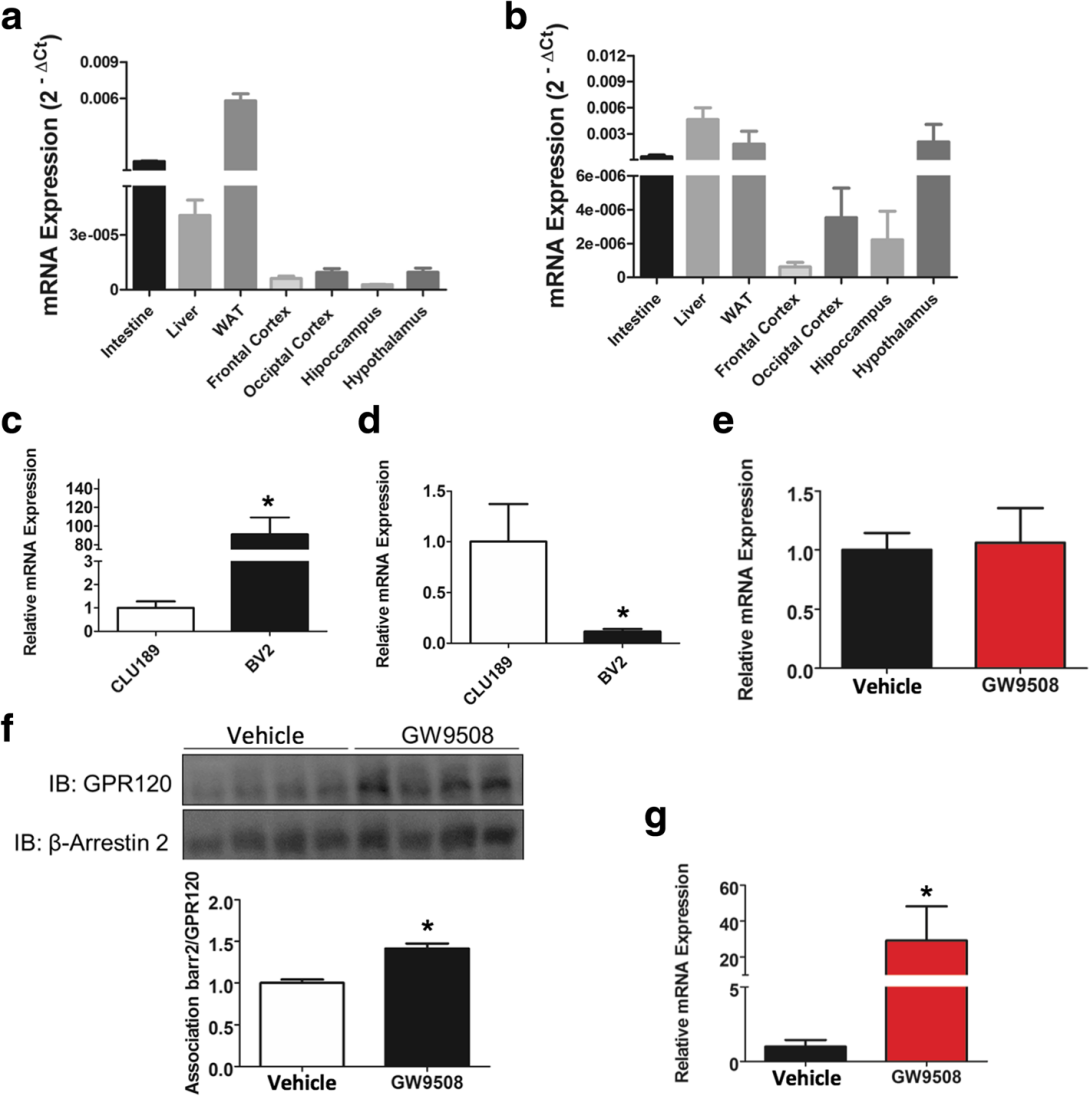
Expression and activation of GPR120 and GPR40. The expression of GPR120 (**a** and **c**) and GPR40 (**b** and **d**) transcripts were evaluated by real-time PCR in samples collected from the small intestine, liver, white adipose tissue (WAT), frontal cortex, occipital cortex, hippocampus, and hypothalamus (**a** and **b**) of lean Swiss mice or in samples prepared from the mHypoA 2/29 CLU189 and BV2 cell lines (**c** and **d**). In **e–g**, lean Swiss mice were subjected to intracerebroventricular cannulation and treated with a single dose of GW9508 (2.0 μl, 1.0 mM); samples were collected after 90 min for the evaluation of GPR120 (*E*) or GPR40 (**g**) transcript expression by real-time PCR. In addition, protein samples were utilized in immunoprecipitation experiments using an anti-β-arrestin-2 antibody; immunoprecipitates were separated by SDS-PAGE, transferred to nitrocellulose membranes, and immunoblotted (IB) with GPR120 or β-arrestin-2 antibodies (**f**). In all experiments, *n* = 5. In *C* and *D*, *p < 0.05 vs. CLU189; in *E* and *F*, *p < 0.05 vs. vehicle

The expression and cellular localization of GPR120 and GPR40 were evaluated in the hypothalamus of male, lean Swiss mice by indirect immunofluorescence staining. As depicted in Fig. 1a–c, the majority of the cells that expressed the microglia marker, mannose receptor, also expressed GPR120 (Fig. 1c). Conversely, we detected no co-localization of GPR120 with either NPY (Fig. 1a) or POMC (Fig. 1b). Figure 2a–c shows that the majority of the cells that expressed NPY (Fig. 2a) and POMC (Fig. 2b) also expressed GPR40, whereas the majority of the cells that expressed the microglia marker, Iba1, did not express GPR40 (Fig. 2c). Because of the localization and shape of GPR120-expressing cells (as depicted in Fig. 1) were similar to that described for tanycytes present in the periventricular area [34, 35], we evaluated the potential co-expression of GPR120 with vimentin (Fig. 3a), a general marker of tanycytes, and IGFBP2 (Fig. 3b), a specific marker for β1-tanycytes [34, 35]. There was only a minor co-localization of GPR120 and vimentin in the region of the junction between the medium eminence and the arcuate nucleus (Fig. 3a). However, no co-localization was observed between GPR120 and IGFBP2 (Fig. 3c). Next, the relative transcript amounts of both GPR120 and GPR40 were evaluated by real-time PCR in samples from distinct tissues of the body. As depicted in Fig. 4a, GPR120 was predominantly expressed in the intestine and white adipose tissue, whereas its levels were low in the central nervous system and similar across the regions tested: frontal cortex, occipital cortex, hippocampus, and hypothalamus. Conversely, the transcript levels of GPR40 (Fig. 4b) were as high in the hypothalamus as in the intestine, liver, and adipose tissue, whereas in the other brain regions tested, its expression was considerably lower. Finally, we evaluated the expressions of GPR120 (Fig. 4c) and GPR40 (Fig. 4d) transcripts in a hypothalamic neuronal cell line, CLU189 [36], and in a microglial cell line, BV2 [37]. Consistent with the findings in the mice, CLU189 cells expressed higher relative amounts of GPR40 compared to GPR120, whereas the opposite occurred in BV2 cells.

### The activation of hypothalamic GPR120 and GPR40 in lean mice

**Fig. 5:**
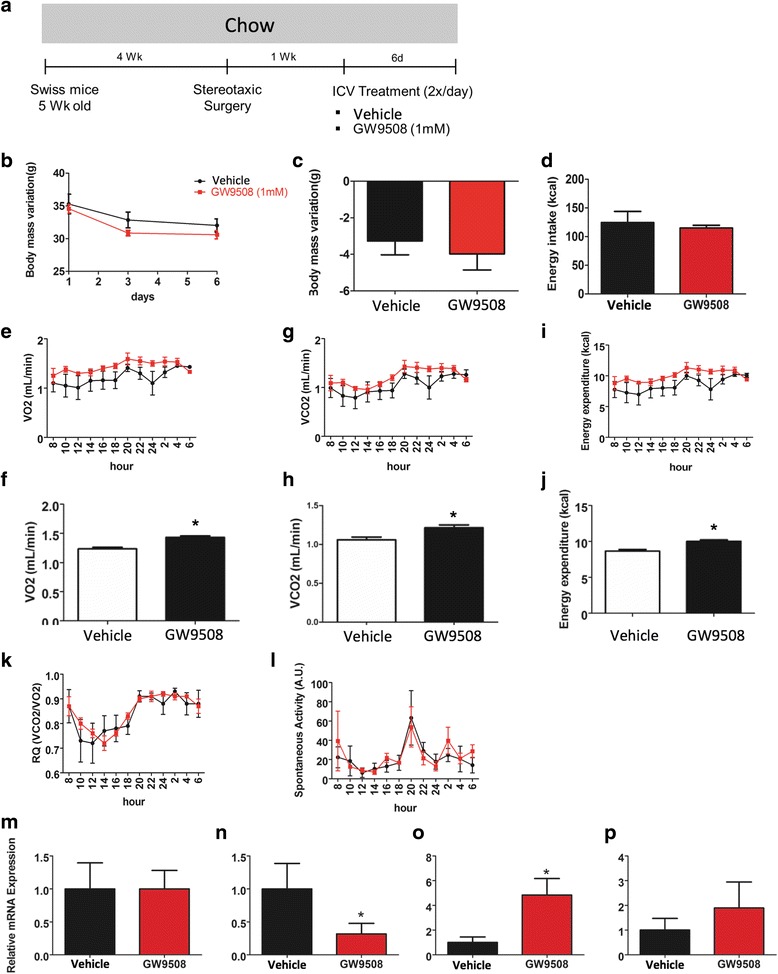
Intracerebroventricular treatment with GW9508 in lean mice. Five-week-old Swiss mice were included in the study and were fed on chow for 4 weeks before intracerebroventricular (icv) cannulation; after 1 week, mice were randomly selected for either vehicle (2.0 μl) or GW9508 (2.0 μl, 1.0 mM) icv treatment twice a day for 6 days (**a**). Body mass (**b** and **c**) and caloric intake (**d**) were measured during the experimental period. At the end of the experimental period, some mice were subjected to indirect calorimetry and the determination of spontaneous physical activity, resulting in the values for O_2_ consumption (*E*–*F*), CO_2_ production (**g–h**), energy expenditure (**i–j**), respiratory quotient (**k**), and spontaneous physical activity (**l**). At the end of the experimental period, the hypothalamus was dissected, and RNA was extracted for real-time PCR determination of transcript levels of TNF-α (**m**), IL1β (**n**), IL10 (**o**), and IL6 (**p**). In all experiments, *n* = 5; *p < 0.05 vs. vehicle

GW9508 is a synthetic agonist that activates GPR120 and GPR40 with EC_50_ values of 2.2 μM and 47 nM, respectively [26]. Upon hypothalamic delivery, GW9508 was not capable of changing GPR120 transcript expression (Fig. 4e) but promoted its activation through the engagement of β-arrestin-2 (Fig. 4f). In addition, GW9508 stimulated an increase of GPR40 transcript levels (Fig. 4g). Next, we treated lean mice with two daily doses of GW9508-delivered icv for 6 days (Fig. 5a). The treatment did not cause a significant change in body mass (Fig. 5b, c) or in spontaneous caloric intake (Fig. 5d). However, mice treated with the compound exhibited significantly increased O_2_ consumption (Fig. 5e, f) and CO_2_ production (Fig. 5g, h), resulting in increased energy expenditure (Fig. 5i, j) in the absence of any modifications of RQ (Fig. 5k) or spontaneous physical activity (Fig. 5l). Interestingly, icv GW9508 induced a reduction of the hypothalamic expression of IL1β (Fig. 5n) and an increase of IL10 (Fig. 5o), without causing a change in TNF-α (Fig. 5m) or IL6 (Fig. 5p).

### The activation of GPR120 and GPR40 in obese mice

**Fig. 6:**
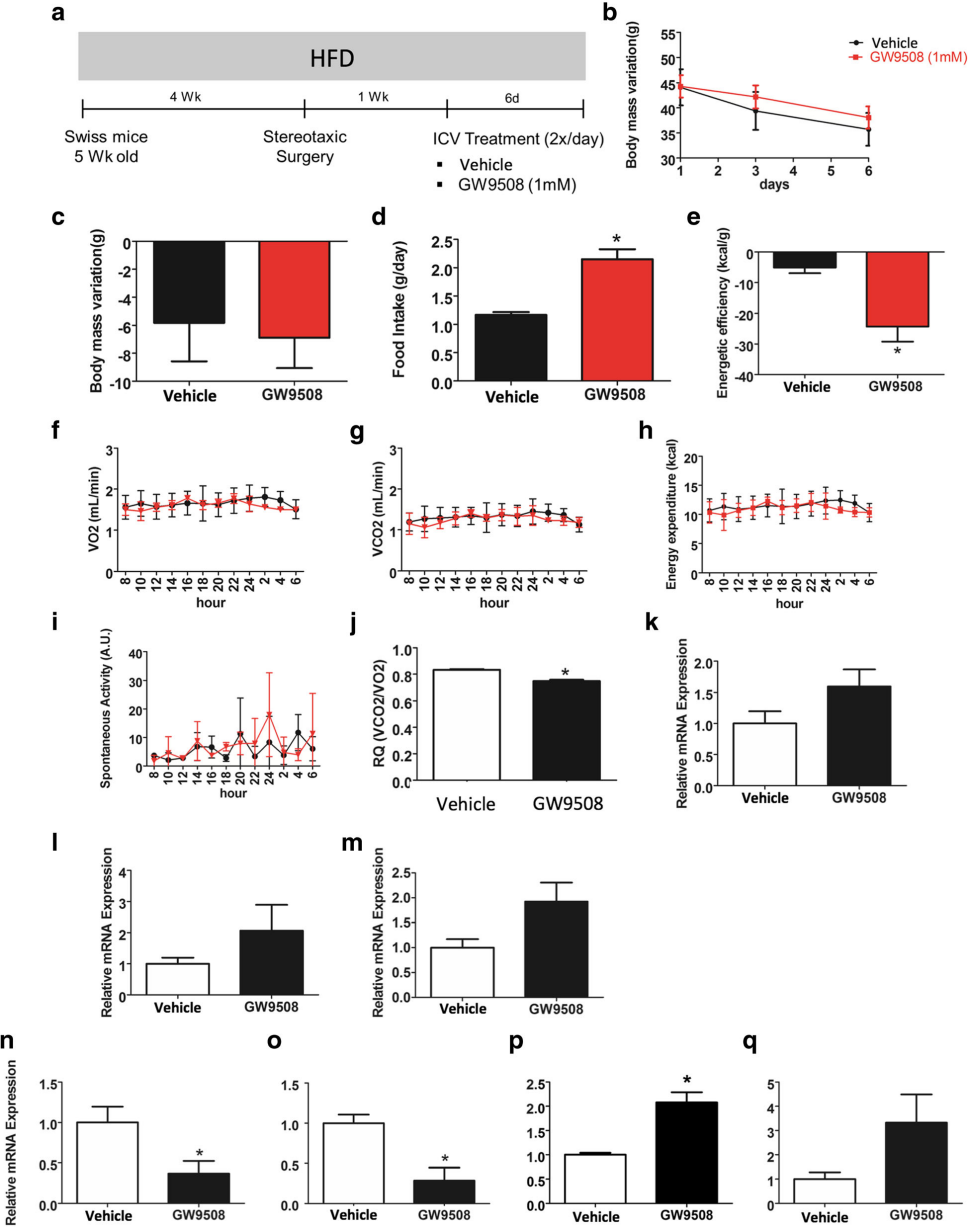
Intracerebroventricular treatment with GW9508 in obese mice. Five-week-old Swiss mice were included in the study and were fed on a high-fat diet (HFD) for 4 weeks before intracerebroventricular (icv) cannulation; after 1 week, mice were randomly selected for either vehicle (2.0 μl) or GW9508 (2.0 μl, 1.0 mM) icv treatment twice a day for 6 days (**a**). Body mass (**b** and **c**), caloric intake (**d**), and energy efficiency (**e**) were measured during the experimental period. At the end of the experimental period, some of the mice were subjected to indirect calorimetry and the determination of spontaneous physical activity, resulting in the values for O_2_ consumption (*F*), CO_2_ production (**g**), energy expenditure (**h**), spontaneous physical activity (**i**), and respiratory quotient (**j**). RNA samples were obtained from the brown adipose tissue for real-time PCR determination of the transcript expression of UCP1 (**k**), PGC1α (**l**), and cytochrome c (**m**). In addition, the hypothalamus was dissected, and RNA was extracted for real-time PCR determination of the transcript levels of TNF-α (**n**), IL1β (**o**), IL10 (**p**), and IL6 (**q**). In all experiments, *n* = 5; *p < 0.05 vs. vehicle

Male Swiss mice were fed on a HFD for 4 weeks and then subjected to icv cannulation for treatment with GW9508 twice a day for 6 days (Fig. 6a). GW9508 produced no change in body mass (Fig. 6b, c) but increased caloric intake (Fig. 6d), resulting in reduced energy efficiency (Fig. 6e). These changes were not accompanied by modifications of O_2_ consumption (Fig. 6f), CO_2_ production (Fig. 6g), energy expenditure (Fig. 6h), or spontaneous physical activity (Fig. 6i). However, RQ was reduced (Fig. 6j), and there were trends toward increased expression of UCP1 (Fig. 6k), PGC1α (Fig. 6l), and cytochrome c (Fig. 6m) in the brown adipose tissue. Moreover, GW9508 reduced hypothalamic TNF-α (Fig. 6n) and IL1β (Fig. 6o), accompanied by increased IL10 (Fig. 6p) without a change in IL6 (Fig. 6q).

### Lentiviral targeting of GPR120 in obese mice

**Fig. 7:**
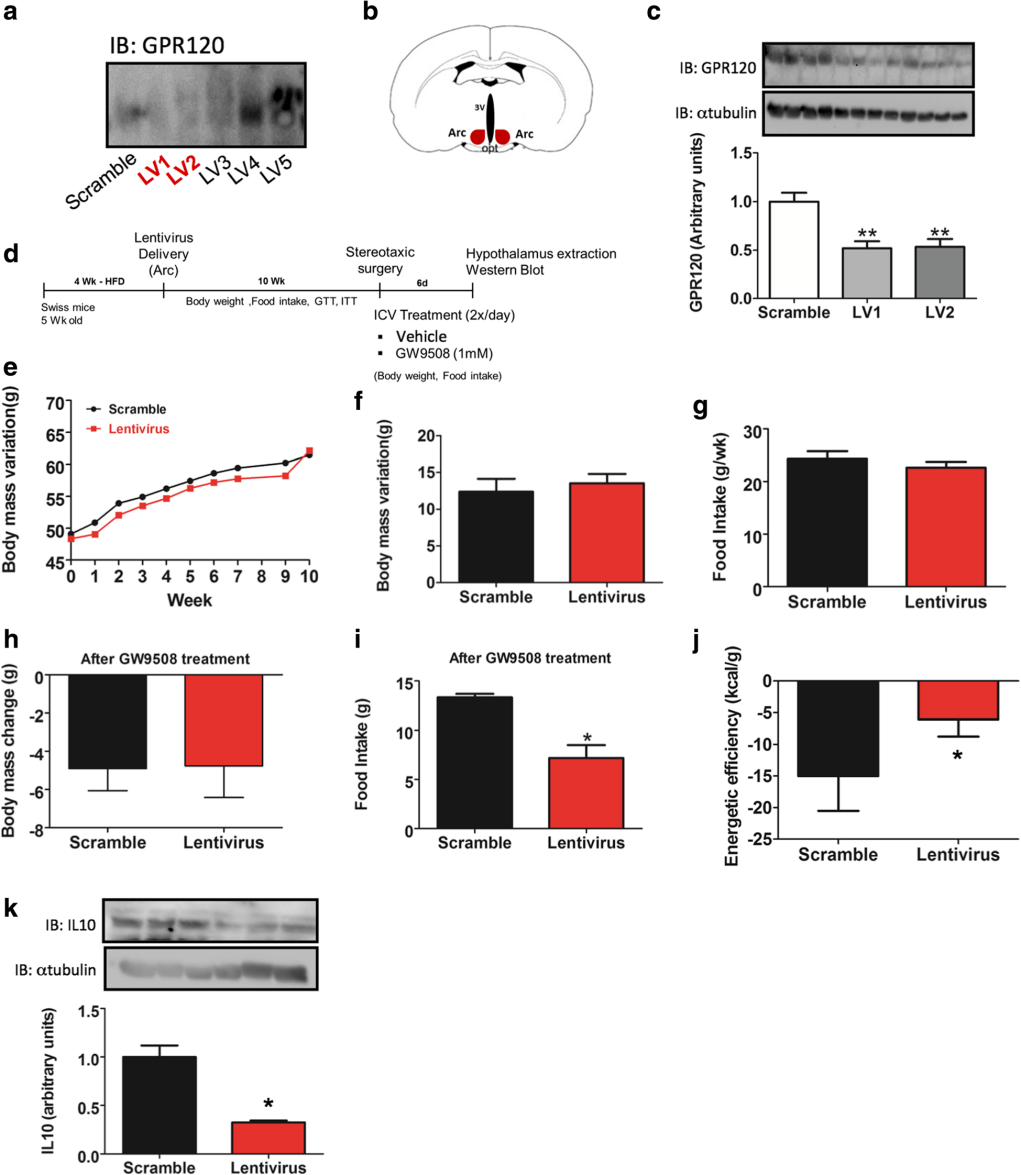
Lentiviral targeting of GPR120. In **a**, the mHypoA 2/29 CLU189 cell line was transfected with lentivirus containing five distinct (LV1–LV5) sequences of shRNA targeting GPR120 and, in addition, a scrambled control. Protein extracts were prepared and subjected to separation by SDS-PAGE, then transferred to a nitrocellulose membrane and blotted with anti-GPR120 antibody. The sequences LV1 and LV2 were utilized in in vivo experiments and were injected bilaterally into the arcuate nucleus (**b**). 7 days after LV1 or LV2 injection, hypothalami were dissected and used for protein extraction. Samples were subjected to separation by SDS-PAGE, transferred to nitrocellulose membranes and immunoblotted (IB) with an antibody against GPR120 (**c**). Swiss mice were fed for 4 weeks on a high-fat diet (HFD) and then injected with LV1 or the scrambled control construct into the arcuate nucleus bilaterally; after 10 weeks, the mice were subjected to intracerebroventricular cannulation and following a recovery period of 7 days, subjected to treatment with GW9508 (2.0 μl, 1.0 mM) twice a day for 6 days (**d**). Body mass (**e** and **f**) and caloric intake (**g**) were determined during the 10 weeks that followed LV1/scramble injection (before GW9508 treatment). Body mass (**h**), caloric intake (**i**), and energy efficiency (**j**) were evaluated during the 6 days that followed GW9508 treatment. At the end of the experimental period, IL10 protein expression was determined in extracts from the hypothalamus (**k**). In **c** and **k**, loading controls were obtained by reprobing the membranes with anti-α-tubulin antibody. In *A*, the results are representative of three independent experiments. In *C*, *n* = 4; *p < 0.05 vs. scramble. In *E*–*K*, *n* = 5; *p < 0.05 vs. scramble

Lentiviruses harboring five different shRNA sequences were employed in an attempt to inhibit the expression of GPR120. As depicted in Fig. 7a, lentiviruses 1 and 2 (LV1 and LV2) were the most effective in reducing GPR120 expression in CLU189 cells. Bilateral injections of either LV1 or LV2 into the arcuate nucleus (Fig. 7b) resulted in the reduction of GPR120 expression (Fig. 7c). Obese mice were then treated with LV1 via bilateral injection into the arcuate nucleus and followed up for 10 weeks before undergoing icv cannulation for treatment with GW9508 for 6 days (Fig. 7d). During the 10 weeks before GW9508 treatment, mice injected with LV1 presented no changes in body mass (Fig. 7e, f) or caloric intake (Fig. 7g). Glucose and insulin tolerance tests were performed but revealed no differences between the groups (data not shown). Following GW9508 treatment, there was no change in body mass (Fig. 7h), but caloric intake was reduced (Fig. 7i), resulting in higher energy efficiency (Fig. 7j). In addition, the hypothalamic expression of IL10 was significantly reduced (Fig. 7k).

### The use of specific synthetic agonists of GPR120 and GPR40 in obese mice

In the last part of the study, we employed two synthetic agonists recently developed to specifically activate either GPR120 or GPR40. TUG1197 activates only GPR120, with a pEC_50_ of 6.32 (inactive on GPR40), whereas TUG905 activates only GPR40, with a pEC_50_ of 7.03 (inactive on GPR120) [38]. The capacity of either compound to reduce LPS-induced activation of IKK was determined in CLU189 (Additional file 1: Figure S2A) and BV2 (Additional file 1: Figure S2B) cells in parallel with GW9508. As shown in Additional file 1: Figure S2A and B, TUG905 was effective in reducing IKK activity in either cell type, whereas TUG1197 was capable of reducing IKK activity only in BV2 cells. GW9508 treatment resulted in a trend to reduce the activation of IKK in BV2 (*p* = 0.07) but not in CLU189, probably because of the low expression of GPR120 in this cell line. Next, obese mice were treated icv with either compound for 6 days (Additional file 1: Figure S2C). TUG1197 had no effect on body mass (Additional file 1: Figure S2D) and caloric intake (Additional file 1: Figure S2E); however, it exerted potent anti-inflammatory activity, reducing TNF-α (Additional file 1: Figure S2F) and IL1β (Additional file 1: Figure S2G) while increasing IL10 (Additional file 1: Figure S2H) and IL6 (Additional file 1: Figure S2I) in the hypothalamus. TUG1197 had no significant effect on the expression of NPY (Additional file 1: Figure S2J) and POMC (Additional file 1: Figure S2K). Conversely, TUG905 reduced body mass (Additional file 1: Figure S2D) and exhibited a trend toward reducing caloric intake (Additional file 1: Figure S2E). TUG905 had no effect on TNF-α (Additional file 1: Figure S2F) and IL1β (Additional file 1: Figure S2G) but induced an increase of IL10 (Additional file 1: Figure S2H) and IL6 (Additional file 1: Figure S2I) in the hypothalamus. In addition, TUG905 had no effect on the transcript levels of NPY (Additional file 1: Figure S2J) but increased POMC (Additional file 1: Figure S2K).

## Discussion

The beneficial effects of dietary PUFAs were first reported in epidemiological studies showing that Inuit and Mediterranean populations are protected from cardiovascular disease due to the consumption of fish and olive oil, which are rich in ω3 and ω9 PUFAs, respectively [39–41]. Mechanistic studies performed during the last 40 years have shown that at least a portion of the favorable metabolic effects of PUFAs is due to their anti-inflammatory actions. There are different anti-inflammatory pathways that can be induced by PUFAs. ω3 fatty acids can compete with arachidonate for 5-LOX and COX2, which catalyze the synthesis of eicosanoids [42, 43]. Variations in the rates of ω3 and ω6 availability can shift the amounts of the final products of this synthetic pathway [44]. When ω6 predominates, pro-inflammatory prostaglandins, leukotrienes, prostacyclins, and thromboxanes are produced [44, 45]. Conversely, upon preferential ω3 availability, the resulting eicosanoids, including protectins, resolvins, and lipoxins, are either less pro-inflammatory or are anti-inflammatory [44–46]. Transcriptional regulation is yet another mechanism underlying the anti-inflammatory actions of PUFAs [47]. DHA can activate PPARγ, leading to the inhibition of inflammatory gene expression [48]. In addition, PPARγ can also inhibit NFκB activity by inducing the expression of IκBα [49].

More recently, new anti-inflammatory mechanisms have been attributed to PUFAs. These findings emerged mostly due to the work exploring the properties of the previously orphan receptors, GPR120, and GPR40 [20, 25]. Because of the potential use of agonists for these receptors as therapeutic tools for metabolic diseases, we decided to evaluate their presence and action in the hypothalamus of obese rodents.

Studies have shown that the consumption of dietary fats, particularly long-chain saturated fatty acids, induces the activation of site-specific inflammation in the hypothalamus [32, 50–54]. At least three distinct mechanisms seem to be involved in the activation of the inflammatory response: TLR4 activation, PKC-theta activation and ERS [32, 50–55]. Pharmacological and genetic approaches used to dampen the inflammation mediated by each of these mechanisms have produced encouraging results with respect to the control of body adiposity and whole-body metabolism [32, 50–55]. Previously, we have shown that PUFAs in the diet or injected directly into the hypothalamus can reduce hypothalamic inflammation and can improve the metabolic phenotype [19]. In addition, PUFAs can induce hypothalamic neurogenesis of predominantly POMC neurons [56]. With these results in mind, we hypothesized that at least a portion of the effects of PUFAs in the hypothalamus could be mediated by GPR120 and GPR40.

Accordingly, in the first part of the study, we showed that both receptors are expressed in the hypothalamus. GPR40 levels were considerably higher than GPR120 levels and were similar to the levels expressed in peripheral tissues, such as adipose tissue, intestine, and the liver. Previous studies have reported the presence of high levels of GPR40 in the brain [25]; however, the majority of the work has been focused on the characterization of its role in hippocampal neurogenesis [28, 57]. There is one previous study showing that GPR40 is present in neurons of the hypothalamus, but the type of neuron was not explored [58]. The study showed an involvement of hypothalamic GPR40 in the response to inflammatory chronic pain [58]. In addition, we showed that inhibiting hypothalamic GPR40 reduces PUFA-induced neurogenesis [56]. Here, we confirmed that GPR40 is present predominantly in hypothalamic neurons, and we expanded this characterization by demonstrating that both NPY and POMC neurons express the highest levels of this receptor.

Regarding GPR120, we showed that it is also present in the hypothalamus; however, the levels of transcript were much lower than in peripheral tissues involved in metabolic activity, such as intestine and adipose tissue [59]. Nevertheless, the levels in the hypothalamus were not that different from the levels in other regions of the brain. The fact that most hypothalamic GPR120 is expressed in microglia may explain why the expression levels were in general quite low. One interesting aspect of the immunofluorescence studies used to characterize the cellular distribution of GPR120 is the fact that some cells expressing GPR120 in the periventricular region nearby the interface between the medium eminence and the arcuate nucleus presented projections in a shape very similar to tanycytes [34, 35]. Because of that, we performed staining with vimentin, which is expressed in different types of tanycytes and IGFBP2, which is expressed in β1-tanycytes. Our results showed only a limited co-localization of GPR120 with vimentin, and, as compared with the staining for mannose receptor, we can conclude that most of GPR120 is expressed in microglia. Our group has published the only previous study reporting the presence of GPR120 in the hypothalamus; however, in that study, we did not explore its cellular distribution or its functional relevance [19]. Another study has shown that GPR120 is expressed in a hypothalamic neuronal cell line and can control inflammation [60].

We initially used a non-specific agonist, GW9508, to evaluate the impact of combined GPR120/GPR40 activation in the hypothalamus. There were few differences in the final outcomes of GW9508 treatment when comparing lean versus obese mice, but as a rule, there were improvements in both inflammatory and metabolic parameters. The impact on the regulation of body mass was not as impressive as the effects obtained when other anti-inflammatory approaches are used to dampen obesity-induced hypothalamic inflammation, such as the inhibition of JNK [50], IKK [54], TLR4 [51], or ERS [52]. In fact, the mild effect obtained with the use of GW9508 is consistent with the fact that reducing GPR120 expression with lentivirus-delivered shRNA also resulted in mild phenotypic changes.

As an attempt to define the specific actions of GPR120 and GPR40 in the hypothalamus, we employed two recently developed compounds that exhibit no cross-activity between these receptors. TUG905 acts on GPR40 only, and upon icv treatment, it produced changes predominantly in energy homeostasis, reducing body mass even in the absence of changes in caloric intake. Interestingly, TUG905 increased the levels of POMC without changing the expression of NPY. At first, this could seem unexpected because the majority of the expression of GPR40 in the hypothalamus is found in NPY neurons; however, it has been shown that NPY neurons can modulate POMC production through a plastic connectivity system [61–63].

Conversely, the action of TUG1197 on GPR120 produced no change in caloric intake and body mass but reduced hypothalamic inflammation. Considering that reductions of body adiposity have been observed in other studies that used approaches that acted solely on the inflammatory aspect of the hypothalamic dysfunction in obesity [32, 50–54], one could ask why this did not happen with TUG1197. In our opinion, there are two possibilities: either (i) the magnitude of the anti-inflammatory effect was not sufficient to modify energy homeostasis; or (ii) 6 days of treatment was not sufficient to observe the putative ongoing changes in the control of energy homeostasis.

Regardless of the answer, we conclude that both GPR120 and GPR40 are expressed in the hypothalamus, and the combined activation of both receptors results in benefits for the control of whole-body energy homeostasis and hypothalamic inflammation. We propose that at least a portion of the metabolic benefits obtained with the consumption of PUFAs may be due to the activation of hypothalamic receptors.

## Conclusions

**Fig. 8:**
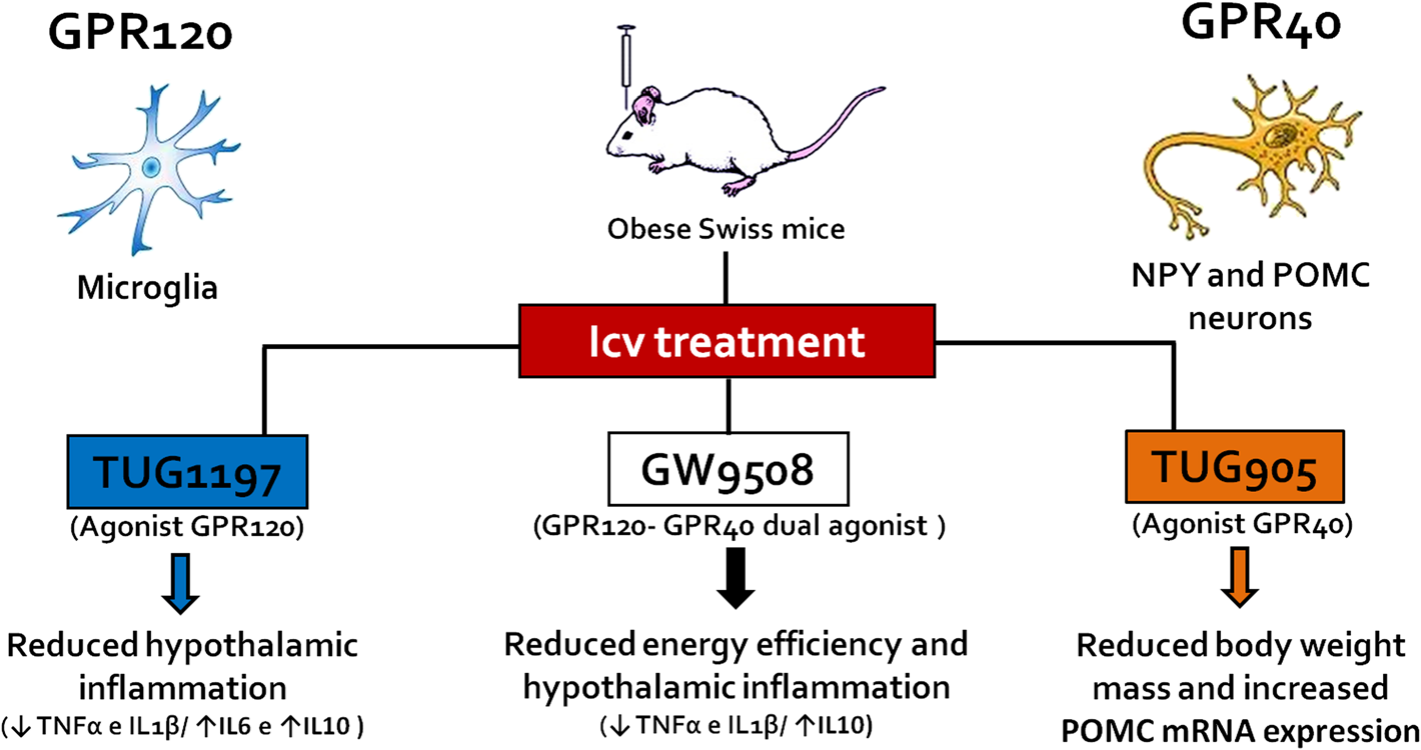
Schematic representation of the main findings of this study. GPR120 and GPR40 are expressed in the hypothalamus of mice; GPR120 is expressed predominantly in microglia whereas GPR40 is expressed predominantly in POMC and NPY neurons. The intracerebroventricular (icv) injection of agonists in mice results in a number of phenotypic changes. TUG1197, the GPR120 agonist, acts predominantly reducing hypothalamic inflammation. TUG905, the GPR40 agonist, acts predominantly reducing body mass and increasing the expression of the anorexigenic precursor, POMC. The use of a compound that acts simultaneously on GPR120 and GPR40, GW9508, results in the best metabolic outcomes, reducing body mass, improving glucose tolerance and reducing hypothalamic inflammation

We show that both receptors are expressed in the hypothalamus; GPR120 is primarily present in microglia, whereas GPR40 is expressed in neurons. We conclude that GPR120 and GPR40 act in concert in the hypothalamus to reduce energy efficiency and to regulate the inflammation associated with obesity. The combined activation of both receptors in the hypothalamus results in better metabolic outcomes than the isolated activation of either receptor alone. Figure 8 presents a schematic view of the main findings of this study.

## Additional file

Additional file 1: Figure S1. Testing the specificity of the antibodies against GPR120 and GPR40. Figure S2 specific activation of GPR120 and GPR40. (PDF 10063 kb)
