# Supplementary material for: Polyunsaturated fatty acid receptors, GPR40 and GPR120, are expressed in the hypothalamus and control energy homeostasis and inflammation
Source: J Neuroinflammation. 2017 Apr 26;14:91. doi: 10.1186/s12974-017-0869-7 (PMC5405534; doi:10.1186/s12974-017-0869-7)

Dragano et al.
Additional file

Figure S1. Testing the specificity of the antibodies against GPR120 and GPR40.

In order to evaluate the specificity of the antibodies against polyunsaturated fatty acid receptors, three experiments were performed. In the first set of experiments, purified GPR40 was bond to a nitrocellulose membrane, which was used in pre-absorption experiments; for that 20 µg of anti-GPR40 antibody was incubated for 24 h with the GPR40 bound to the nitrocellulose membrane and then used in immunoblot experiments against a total protein extract prepared from the neuronal cell line CLU189 separated by SDS-PAGE and transferred to a nitrocellulose membrane. Anti-GPR40 antibody not submitted to the pre-absorption procedure can recognize GPR40 (A); anti-GPR40 submitted to the pre-absorption procedure cannot recognize GPR40 (B); anti-ERK1/2 antibody submitted to the pre-absorption procedure (against GRP40 bound to the nitrocellulose membrane) can recognize ERK1/2 (C). The membrane used in the experiments was stained with Ponceau (D). In the second set of experiments, the neuronal cell line CLU189 was treated with a control lentiviral plasmid (scramble) or an shRNA-encoding lentivirus plasmid against GPR40 (LVGPR40). Whole cell lysates were prepared and protein extracts were employed in immunoprecipitation experiments using either non-immune serum (NIS) or anti-GPR40 antibody (Anti-GPR40). The immuoprecipitates were collected and separated by SDS-PAGE, transferred to nitrocellulose membranes, which were immunobloted with anti-GPR40 antibodies. As depicted in E, a band corresponding to GPR40 is detected only in the samples treated with the control lentivirus and immunoprecipitaded with anti-GPR40 antibody. A similar experiment was performed using the anti-GPR120 antibody, but in this case no specific band could be detected. In the second set of experiments, the microglia cell line BV2 was treated with a control lentiviral plasmid (scramble) or an shRNA-encoding lentivirus plasmid against GPR120 (LVGPR120). Cells were harvested for cDNA preparation and real-time PCR analysis to analysis of GPR120 transcript expression. As shown in F, the treatment with the GPR120 lentivirus resulted in approximately 50% reduction of the expression of the target transcript. Next cells were employed in immunofluorescence experiments to detect the presence of either GPR120 or GRP40. In G, the cells were treated with the control lentivirus and GPR120 was easily detected. In H, in cells treated with the anti-GPR120 lentivirus the expression of GPR120 was considerably reduced. The anti-GPR40 antibody was virtually incapable of detecting antigen in either cell preparation. In A-E, G and H the experiments were repeated three times; inF, n=5. In G and H, cell nuclei were stained with DAPI. The GPR120 and GPR40 antibodies (sc99105 and sc32905, from Santa Cruz Biotechnology) were used with success in other studies: Endocrinology 2015, 156:4033; PLoS One 2012, 7:e30571; Diabetologia 2013, 56:2456; Nutr Metab Cardiovasc Dis 2010, 20:22; BBRC 2010, 403:36; Mol Cell Endocrinol 2008, 294:81.

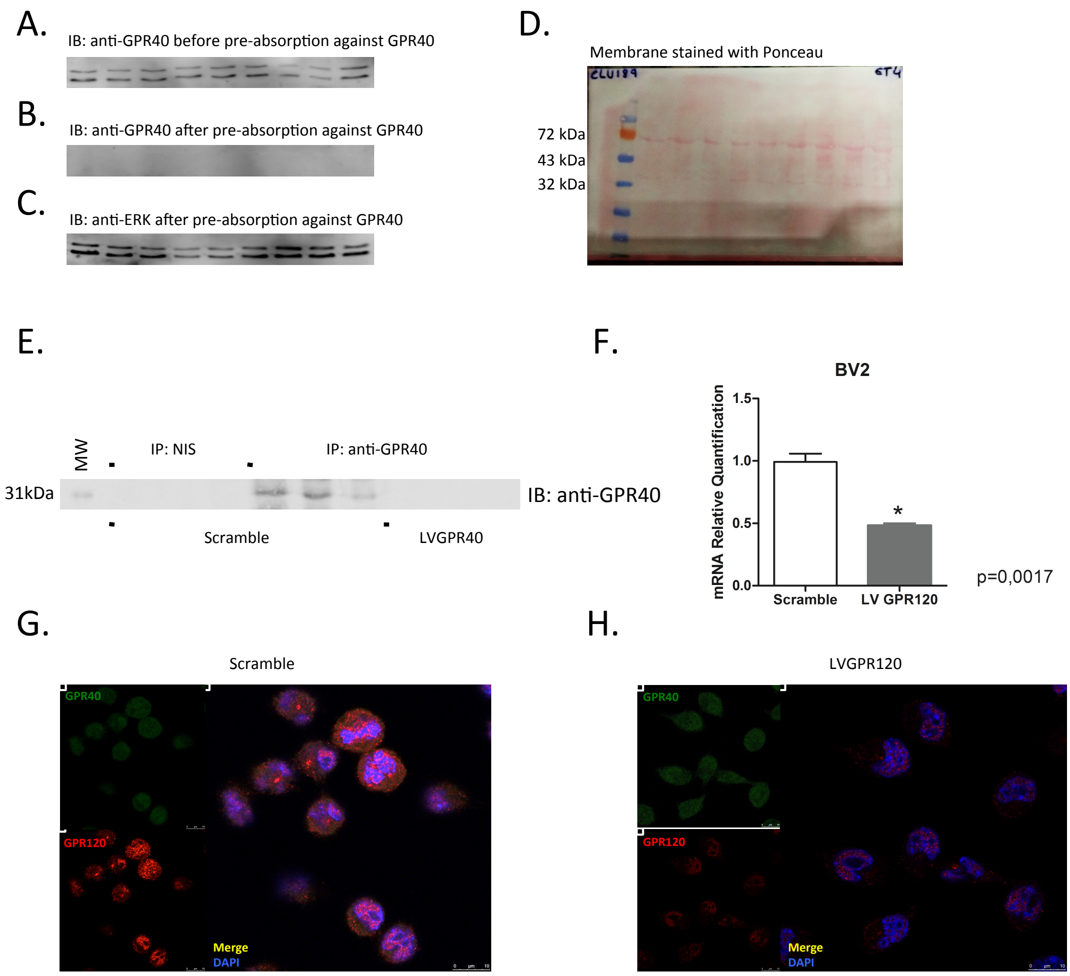
A.
IB: anti-GPR40 before pre-absorption against GPR40
B.
IB: anti-GPR40 after pre-absorption against GPR40
C.
IB: anti-ERK after pre-absorption against GPR40
D.
Membrane stained with Ponceau
72 kDa
43 kDa
32 kDa
E.
MW
IP: NIS
IP: anti-GPR40
31kDa
IB: anti-GPR40
Scramble
LVGPR40
F.
BV2
mRNA Relative Quantification
1.5
1.0
0.5
0.0
*
Scramble
LV GPR120
p=0,0017
G.
Scramble
GPR40
GPR120
Merge
DAPI
H.
LVGPR120
GPR40
GPR120
Merge
DAPI

Figure S2. Specific activation of GPR120 and GPR40. The mHypoA 2/29 CLU189 (A) and BV2 (B) cell lines were treated with lipopolysaccharide (LPS, 100 ng/mL) for 10 min in the presence of either GW9508 (100 μM), TUG1197 (100 μM) or TUG (100 μM), which were added to the culture media 60 min prior to the LPS addition. Protein extracts were prepared and subjected to separation by SDS-PAGE, then transferred to a nitrocellulose membrane and blotted (IB) with anti-phospho-IKK; loading controls were obtained by reprobing the membranes with anti-α-tubulin antibody. The protocol employed for studying the *in vivo* effects of the agonists of GPR120 and GPR40 is presented in C; five-week-old Swiss mice were included in the study and fed on a high-fat diet (HFD) for four weeks before intracerebroventricular (icv) cannulation; after one week, the mice were randomly selected for either vehicle (2.0 μl) TUG1197 (2.0 μl, 1.0 mM) or TUG905 (2.0 μl, 1.0 mM) icv treatment twice a day for six days. Body mass (D) and caloric intake (E) were determined during the treatment period. At the end of the experimental period, hypothalamic RNA was extracted and utilized in real-time PCR to determine the expression of TNFα (F), IL1β (G), IL10 (H), IL6 (I), NPY (J) and POMC (K). In all experiments, n=5. In A and B,*p<0.05 vs. LPS. In F-K, p<0.05 vs. vehicle.

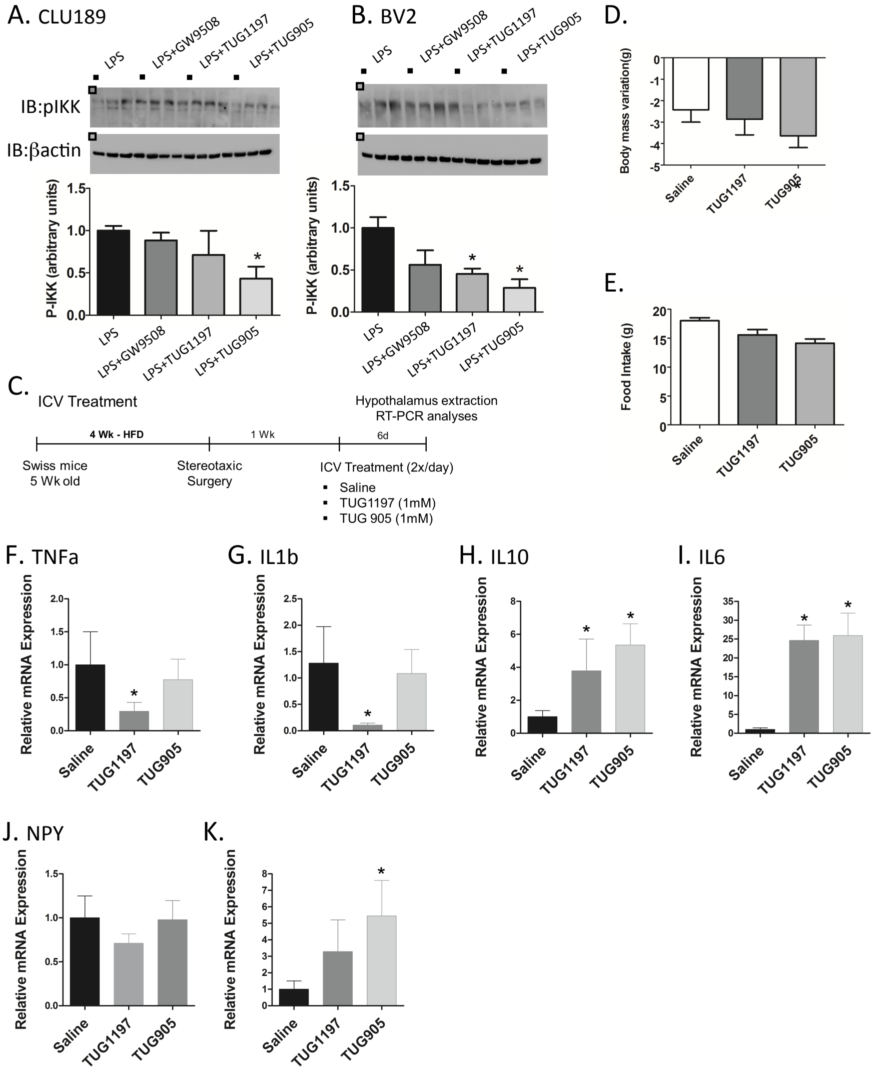

Supplement: Additional file 1: Figure S1. — Testing the specificity of the antibodies against GPR120 and GPR40. Figure S2 specific activation of GPR120 and GPR40. (PDF 10063 kb) [file 12974_2017_869_MOESM1_ESM.pdf]
